# Age-related dysfunctions of the neuroendocrine axes in nonhuman primates with depression-like and anxious behavior

**DOI:** 10.18632/aging.206388

**Published:** 2026-06-11

**Authors:** Nadezhda D. Goncharova

**Affiliations:** 1 Laboratory of Experimental Endocrinology, Kurchatov Complex of Medical Primatology of the National Research Center “Kurchatov Institute”, Adler, Sochi 354376, Russian Federation

**Keywords:** the hypothalamic-pituitary-adrenal axis, the hypothalamic-pituitary-thyroid axis, stress behavior, aging, Insulin resistance

## Abstract

With age, the incidence of stress-related pathologies, in the etiopathogenesis of which endocrine dysfunctions play an important role, increases. This review presents data on the study of age-related features in functioning of the key adaptive neuroendocrine axes (hypothalamic-pituitary-adrenal, HPA axis and hypothalamic-pituitary-thyroid, HPT axis) in individuals that differ in adaptive behavior with an emphasis on experimental studies in nonhuman primates. Studies have shown pronounced age-related differences in the functioning of HPA axis in the animals with depression-like and anxiety behavior (DAB): impairment negative feedback regulation with an increase of corticotropin and cortisol level in the afternoon - night time, a higher response to acute stress exposure, and sensitization of the hypothalamic-pituitary axis. A decrease secretion of thyroxine and its response to thyrotropin-releasing hormone (TRH) or thyrotropin administration with increased sensitivity of the adenohypophysis to TRH were identified in old DAB animals. In addition, older overweight DAB monkeys exhibited increased insulin resistance and reduced insulin and triglycerides secretion. Thus, age-related changes of HPA and HPT axes in DAB monkeys are associated with more pronounced endocrine dysfunctions as compared with young individuals, leading to hormonal imbalance that may contribute to the development of severe age-related pathology.

## Dedication

This review is dedicated to the memory and honor of Dr. Mikhail Blagosklonny, a preeminent scientist in oncology, the biology of aging, longevity and personalized medicine. I am deeply grateful to Mikhail Vladimirovich for supporting my research in the field personalized approaches to the endocrinology of aging, specifically using nonhuman primate models under mild to moderate stress. My previous work was published in Aging in Volume 2, Issue 11. I hope that this new article, which reviews literature on individual differences in age-related neuroendocrine dysregulation as a foundation for stress vulnerability, accelerated aging, and age-related pathology, will be a worthy tribute to Dr. Mikhail Blagosklonny's memory.

## INTRODUCTION

With aging, the frequency of stress-related diseases, including mental, metabolic, endocrine, cognitive, cardiovascular, and neurodegenerative diseases increases dramatically [1–9]. Despite this generalized phenomenon, there are notable individual differences in both vulnerability and resilience to stress and stress-related pathologies [4, 8, 10–12]. In particular, in spite of the abundance of ongoing studies in stress research, the mechanisms underlying individual vulnerability to stress and age-related diseases remain unclear. It is well known that the most important adaptive neuroendocrine systems, the hypothalamic-pituitary-adrenal (HPA) axis and hypothalamic-pituitary-thyroid (HPT) axis, are essential modulators of both the endocrine and behavioral adaptation of the organism to changing environmental factors, and their dysfunction can contribute to the development of various stress-dependent socially significant diseases. The review focuses on individual differences in age-related neuroendocrine dysregulation as a mechanistic basis for stress vulnerability, accelerated aging, and age-related pathology.

The HPA axis, a key adaptive neuroendocrine axis, reacts to all environment stimuli in a non-specific way, activating the secretion of corticotropin-releasing hormone (CRH) and arginine vasopressin (AVP) by the hypothalamic paraventricular nucleus (PVN), which in turn activate the secretion of corticotropin (ACTH) by corticotrophs of the anterior pituitary. ACTH stimulates corticosteroid secretion, mainly cortisol (CORT) and dehydroepiandrosterone (DHEA) with DHEA sulfate (DHEAS) in humans and nonhuman primates, and corticosterone in rodents. Corticosteroids act on specific receptors present in most peripheral tissues and the brain and trigger the metabolic, immune, neuromodulatory, and behavioral changes needed to cope with the impact of the stressors [10–14]. While adequate activation of the HPA axis during acute stress is critical for survival, inadequate activation (too high, prolonged, unpredictable, etc.) leads to hypercortisolemia, which is known to play a pathophysiological role in the development of various stress-related diseases, including reproductive, immune, metabolic, cardiovascular, psychiatric, and neurodegenerative [8, 10–14].

Activation of the HPT axis occurs mainly in response to a decrease in ambient temperature, physical and mental stress and other stimuli. The main component of this axis is the PVN of the hypothalamus, where thyrotropin-releasing hormone (TRH) is produced in specific parvocellular neurons and secreted into the pituitary portal system. The interaction of TRH with specific receptors on thyrotrophs of the anterior pituitary gland induces the release of thyroid-stimulating hormone (thyrotropin, TSH) into the circulation. TSH interacting with receptors on thyroid gland cells stimulates the uptake of molecular iodine from the blood and the synthesis and secretion of thyroid hormones, thyroxine (T4) and triiodothyronine (T3). Through specific cell receptors, thyroid hormones mediate the physiological effects of the HPT axis and regulate the function of this axis at the level of the pituitary and hypothalamus by a negative feedback mechanism. The HPT axis regulates diverse physiological processes such as regulation of lipid metabolism and glucose homeostasis, thermogenesis, cellular repair mechanisms, normal growth, development and differentiation of nerve cells, reproduction, homeostasis of the cardiovascular system [2, 7, 8, 15–17]. Thyroid dysfunction is considered to be associated with type 2 diabetes mellitus [18–20], metabolic syndrome [20–21], fatty liver disease [17, 22], depression [3, 23]. According to the majority of experimental data, the activity of the HPT axis decreases in response to stress exposure [24–26]. The inhibitory effect of stress on the function of the HPT axis is apparently based on the inhibitory effect of increased glucocorticoid concentrations, which can be realized at the level of pituitary thyrotrophs [25–26], the hypothalamic paraventricular nucleus [24–26], and at the level of peripheral conversion of T4 to T3 [27].

The functions of the endocrine systems generally deteriorate during aging both in basal conditions and in response to stress exposure or their specific activation, increasing the risk of age-related disorders [8, 10, 28–33]. For example, it has been found that hypercortisolemia and increased vulnerability to stress exposure are characteristic of age-related dysfunctions of the HPA axis [8, 11, 32–33]. Circulating concentrations of DHEAS peak at about age 25 and then decline gradually with age, falling to childhood concentrations by age 80 in most adults, reflecting a gradual reduction in the size of the zona reticularis [34–35]. The majority of older individuals with elevated TSH concentrations have normal free T4 concentrations, a combination of thyroid test results is known as subclinical hypothyroidism [36]. The insulin resistance and hyperinsulinemia are characteristic for age-related dysfunctions of the pancreatic islet apparatus [5, 8, 30, 37] and are usually associated with thyroid and adrenal dysfunctions [8, 18–20].

However, the type and magnitude of deterioration are not similar in all individuals and appear to be associated with different behavioral characteristics. For example, it has been found that trajectory of age-related decline of plasma DHEA (DHEAS) concentrations vary significantly among individuals, and in population studies, DHEAS concentrations are higher in men than women [8, 38]. A higher activity of the HPA axis has been noted in elderly individuals with depression and in nonhuman primates that exhibit depression and anxiety-like behavior [8, 11–12, 39, 40]. The data presented a potential risk of revealed reduced adaptive capacity the HPA axis under constant lighting in rhesus monkeys for the development of stress-related pathology and accelerated aging, especially in the depression-like/anxiety-like compromised animals [41]. Konstandi and Johnson [42] discussed the data on the activation of the HPA axis during stress and aging that can lead to disruption of hepatic cytochrome P450 (CYP)-dependent drug metabolism with pronounced inter-individual variations in the effectiveness and side effects of standard treatment protocols. The article by Degroote et al. [43] focuses on the study of the relationship between individual differences in the daily activity of the HPA axis in middle-aged and elderly people and coronary heart disease. A growing body of work also points to the regulatory influence of steroid and thyroid hormones on lifespan through modulation of key cellular pathways associated with the main hallmarks of aging and longevity [15, 44–45]. The majority of clinical studies indicate a significant predominance of age-related dysfunctions of HPA and HPT axes in women compared to men and in females compared to male nonhuman primates [1, 8–9, 12, 46], which indicates the importance of sex hormones, and especially estrogens, in age-related impairment of stress reactivity of these neuroendocrine systems.

Тhus, age-related dysfunctions of the neuroendocrine axes form the pathophysiological conditions for the development of age-related pathology. Individual differences in the characteristics and intensity of these age-related endocrine dysfunctions may underlie differences in vulnerability to age-related pathology [40]. Therefore, study of individual features of aging of the key endocrine adaptive systems is important to identify individuals with increased vulnerability to stress and accelerated aging and to develop a personalized approach to the prevention and treatment of stress-related pathology in the elderly. Biomarkers are needed both to identify individuals with increased vulnerability to stress and age-related pathologies and for understanding the mechanisms of individual differences. Dynamic responses (stress tests, circadian profiles, tests with the administration of CRH, AVP, ACTH, TRH, TSH, etc.) are more informative than basal hormone levels as biomarkers of increased vulnerability to stress and age-related pathology [8, 10–11, 31–33].

Since most studies on this problem were carried out in experiments on nocturnal rodents [28, 30, 47, 48], which differ significantly from humans in the functioning of HPA axis, and individual features of age-related changes were assessed, as a rule, only for one of the endocrine systems, mainly HPA axis, it is of considerable interest to simultaneously study the age-related features of the functioning of various parts of the endocrine system in an experiment on an adequate experimental model. Nonhuman primates, by virtue of their genetic, physiological, and behavioral similarities with humans, appear to be the optimal translational model for such research [35, 49–59].

One of the most promising experimental approaches to studying individual characteristics of the aging endocrine system is apparently the study of age-related features of the endocrine system in individuals who differ in the characteristics of higher nervous activity, in particular in behavior under conditions of mild/moderate stress [11, 31, 33, 37, 38, 51, 55, 60–63]. For example, the behavior of rhesus monkeys in an enclosure is characterized by social behavior and groups of animals with huddling and non-huddling behavior are distinguished; cortisol levels were elevated significantly in high huddlers compared with low huddlers and non-huddlers [53]. In addition, it has been demonstrated that in the marmoset monkey the serotonergic and regional brain volume correlates with trait anxiety and high-anxious marmosets showed reduced amygdala serotonin levels, and smaller volumes in a closely connected prefrontal region and the dorsal anterior cingulate cortex [51]. These findings highlight behavioral and neural similarities between trait-like anxiety in monkeys and humans. Low- and high-anxious animals were identified by behavioral responses to a human intruder [51]. Studies by other authors made it possible to distinguish animals (female rhesus monkeys) with standard control (SB) and depression-like and/or anxiety-like behavior (DAB) by studying the behavior of monkeys housed individually [31, 37, 38, 41]. During the 2-week period immediately following the transfer of monkeys from their usual residence, in groups, into the individual metabolic cages, they exhibited considerable orientation and aggressive defensive unconditioned reflexes in response to new living conditions, as well as to the procedure of bleeding. A training procedure with monkeys during 4-week after transporting animals in metabolic cages was sufficient for the elimination of these reflexes in animals with SB, but not in animals with depression-like and anxiety behavior. Behavior following the adaptation period, i.e., during experimentation was distinctly different between groups, and it was considered by the authors to be each animal's individual response to mild stress induced by the procedural stress [31, 38, 41]. The data on the polymorphism of the behavioral response of nonhuman primates to mild stress and their spontaneous anxious and depressive behavior are confirmed by data from other authors [53, 61, 64].

This review presents data on the study of age-related features in functioning of the key adaptive neuroendocrine systems, HPA and HPT axes, in individuals that differ in adaptive behavior with an emphasis on experimental studies in nonhuman primates. This review may be useful for researchers and clinicians interested in the problems of identifying individuals with increased vulnerability to stress and accelerated aging and developing a personalized approach to the prevention and treatment of stress-related pathology in the elderly.

## Hypothalamic – pituitary - adrenal axis

An increasing number of studies, both clinical [45, 62, 65–68] and experimental [11, 33, 38, 48, 53, 60, 61, 69], indicate an association between the characteristics of an individual’s HPA axis response to stress and the characteristics of his adaptive behavior. However, most of these studies concerned mainly the young period of individuals' lives and did not study the mechanisms of age-related changes in HPA axis function depending on behavioral characteristics [60, 61, 66, 68, 69]. In addition, most of them assessed HPA axis response to stress without taking into account the circadian rhythm of HPA axis activity under basal conditions. At the same time, a number of publications noted the phenomenon of dependence of stress reactivity of the HPA axis on the time of day [10, 11, 45, 70–72]. In addition, many authors believe that age-related disturbances of the circadian rhythms of the HPA axis play an important role in the development of age-related diseases [11, 71, 73, 74]. Individual differences in the regulation of the HPA axis via a negative feedback mechanism based on mineralocorticoid receptors (MRs) have also been noted in humans and primates, showing a strong time-of-day dependence [31, 70, 75].

In the context of studying the age-related features of the HPA axis in individuals with various adaptive behavioral characteristics, the results of systematic studies conducted at the Adler Primatology Center using special technologies are of considerable interest. Their approach to the problem of the relationship between behavior and endocrine system was based on observations of nonhuman primates (female rhesus monkeys), usually living in enclosures or cages designed for group housing, and for the experiments moved in individual metabolic cages. It turned out that after a 4-week adaptation period, the animals behave differently. The specifics of keeping animals, their physical condition and behavior, which underlie the formation of a particular behavioral group, were described in detail earlier [31, 33, 38].

Three main types of primate behavior can be distinguished: standard (SB, healthy, control), depression-like and anxiety-like (DAB), and aggressive [31, 33, 38, 41]. Since individual adaptive behavior of animals is quite stable and characterizes the features of the higher nervous activity of individuals, in the regulation of which the endocrine system plays an important role, the authors attempted to establish a relationship between individual characteristics of stress behavior and the functioning of the endocrine system. It turned out that all the above-mentioned types of behavior differ, first of all, in the functioning of the HPA axis - a key adaptive neuroendocrine axis, the dysfunction of which plays a central role in the pathophysiology of stress and age-related pathologies.

First, they identified differences in the functioning of HPA axis in these behavioral types at aging under basal conditions, namely: in the secretion of DHEAS, the cortisol/DHEAS (CORT/DHEAS) molar ratio, the CORT plasma levels in the evening, and the amplitude of the circadian rhythm of CORT. Thus, the minimum values of DHEAS concentration and the maximum values of the CORT/DHEAS molar ratio were observed in old females with DAB behavior. Statistically significant differences were also noted between animals of different behavioral groups in relation to cortisol concentrations in the evening. It was significantly higher at 21:00, and the amplitude of the CORT circadian rhythm was correspondingly lower in animals with DAB compared to animals with other types of behavior [38]. A decrease in basal DHEA (DHEAS) levels and an increase in the cortisol/DHEAS ratio are characteristic of aging humans and primates [8, 10, 35, 76, 77].

They also hypothesized that different behavioral patterns in female rhesus monkeys may differ in the HPA axis response to stress exposure. In developing experimental approaches to assessing possible individual differences in HPA axis response to stressors, they relied on the results of their previous studies on the circadian rhythm of the HPA axis response to acute stress exposure (ASE, restraint for 2 hours) in young monkeys without taking into account their behavioral characteristics [11, 72, 78]. They demonstrated a significantly higher increase in ACTH and CORT levels in young female rhesus monkeys when the stress was applied at 15:00 then at 09:00. At the same time, they showed a smoothing of the circadian rhythm of plasma ACTH and CORT in old female monkeys and pronounced age-related differences in the stress reactivity of HPA axis at 15:00 with a lower reaction in old animals [11, 72, 78].

Based on the obtained data that the response of the HPA axis to ASE depends on the time of day, an attempt was made to identify possible individual differences in the stress reactivity of the HPA axis when exposed to stress at 15:00. It turned out, no differences in ACTH and CORT response to ASE were detected between young animals with DAB and SB [33], but intergroup differences were found in them in ACTH response to CRH and AVP tests, with a higher response in young DAB animals [79, 80]. These phenomena are illustrated in Figures 1, 2, respectively. These data indicate that, despite the absence of intergroup differences in HPA axis response to ASE, there are intergroup latent differences in the functioning of the HPA axis in young animals with DAB.

**Figure 1:**
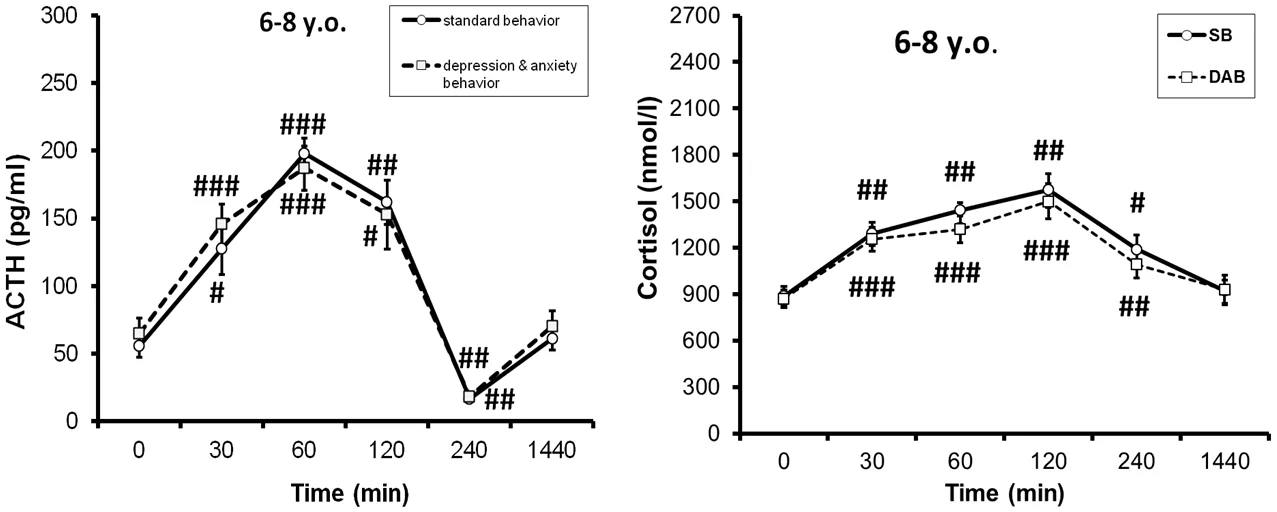
**Dynamics of ACTH and cortisol concentrations (mean±S.E.M) in peripheral blood plasma of young female rhesus monkeys with different types of behavior in response to acute stress exposure (restraint for 2 hours, begun at 15:00h).** # p<0.05, ## p<0.01, ### p<0.001– vs. basal values (0 min). Adapted with permission in [33]. Copyright. Elsevier.

**Figure 2:**
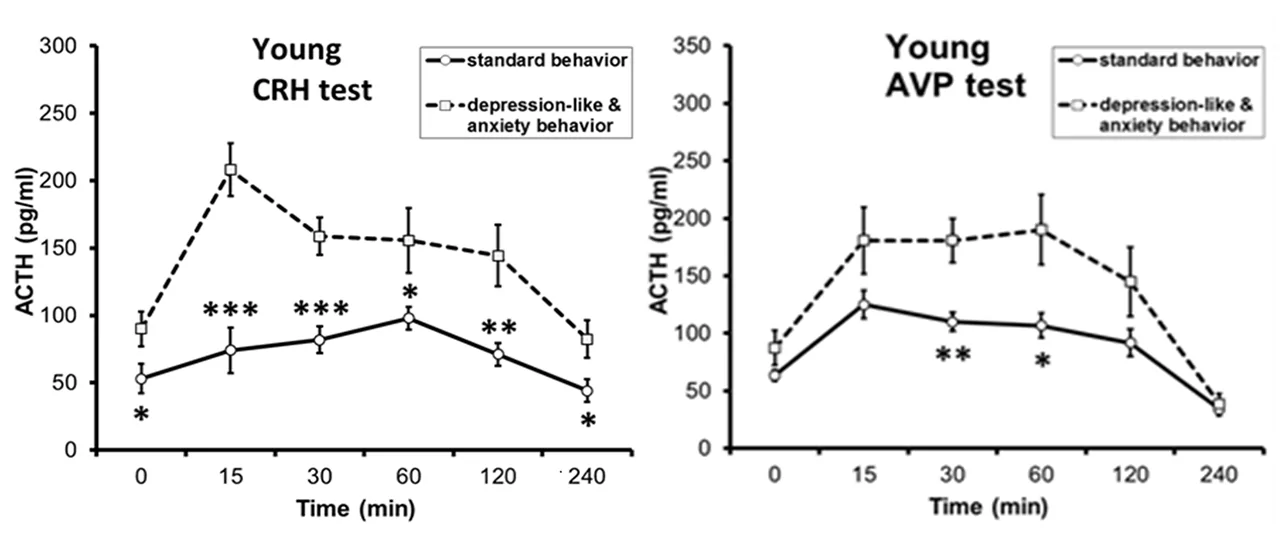
**Dynamics of plasma ACTH level in response to functional tests with administration of CRH and AVP to young female rhesus monkeys with different types of behavior (mean±S.E.M; begun at 15:00h).** *p < 0.05; **p < 0. 01; **p < 0.001 – vs. relative values in animals with DAB.

Unlike young animals, pronounced intergroup differences in ACTH response to ASE were revealed in old female rhesus monkeys with higher response in animals with DAB [33]. The identified intergroup differences in the ACTH response to ASE in old animals are apparently due to the age-related differences in the ACTH response to ASE in animals with DAB and SB. Indeed, as the experimental data showed, old animals with SB are characterized by a marked decrease in the ACTH response to ASE compared to young animals. At the same time, for old animals with DAB, the ACTH response to ASE was mainly not significantly changed or increased compared to young animals with DAB [33]. In contrast to ACTH, the rise in CORT levels in response to ASE in old DAB animals was lower than in old SB animals, presumably due to compensatory desensitization of corticocytes following central sensitization of the HPA axis [33]. Figure 3 shows the differences in ACTH and CORT responses to ASE in aged female rhesus monkeys with SB and DAB. However, the CORT/DHEAS molar ratio was higher than in SB animals, i.e., DAB females developed relative hypercortisolemia [33]. Signs of increased secretory activity of corticotrophs of the anterior pituitary gland were observed in pathomorphological material from the majority of elderly cadavers (men over 70 years of age) [81].

**Figure 3:**
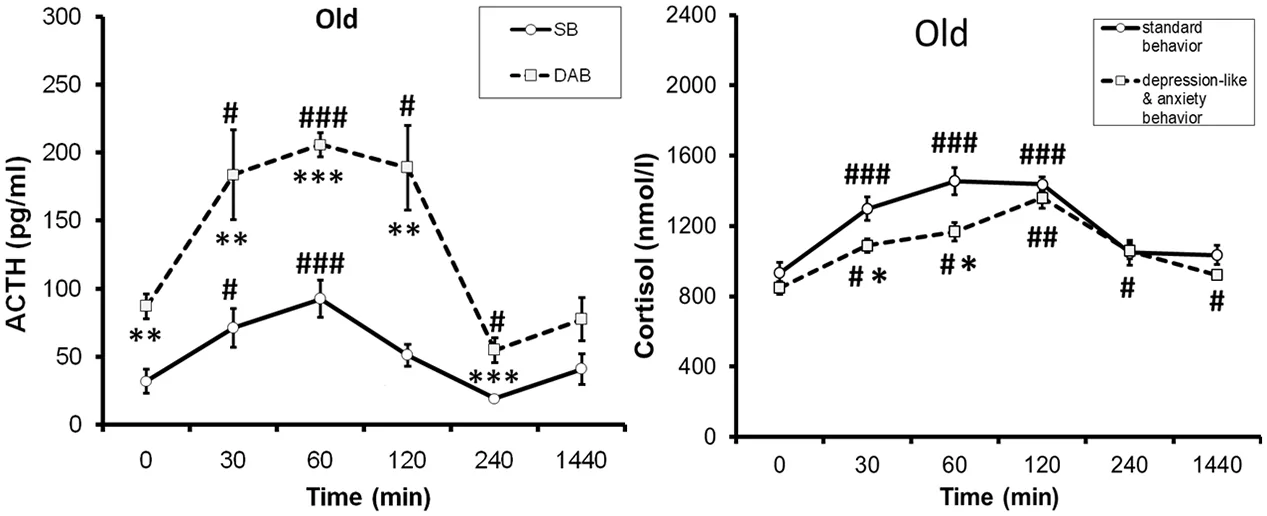
**Dynamics of ACTH and cortisol concentrations (mean±S.E.M) in peripheral blood plasma of old female rhesus monkeys with different types of behavior in response to acute stress exposure (restraint for 2 hours, begun at 15:00).** # p < 0.05, ## p < 0.01, ### p < 0.001– vs. basal values (0 min). *p < 0.05; **p < 0. 01; **p < 0.001 – vs. relative values in animals with SB. Adapted with permission in [33]. Copyright. Elsevier.

What are the mechanisms of intergroup differences in ACTH response to acute psycho-emotional stress in old female *Macaca mulatta*? In order to answer this question, a study was initiated to investigate the age-related features of the ACTH response to functional tests with CRH and AVP in female rhesus monkeys depending on their behavioral characteristics. As already mentioned above, intergroup differences were found in reaction of ACTH to CRH or AVP tests in young mature animals with higher response in DAB animals. It was further found that with aging in all animals, regardless of behavior, the ACTH response to the CRH test does not undergo significant changes and remains higher in old animals with DAB compared to old animals with SB [79]. At the same time, the ACTH response to the AVP test had a character similar to age-related changes in the ACTH response to the ASE, i.e., it was associated with behavioral characteristics. It decreased with aging in animals with SB, but increased in animals with DAB [10, 80, 82]. The presented experimental data are illustrated in Figure 4. Research with parallel determination of ACTH and AVP levels under stress conditions (restraint for 2 hours or insulin-induced hypoglycemia), as well as with the vasopressin V1b receptor antagonist (SSR 149415, Nelivaptan, “Axon”, Netherlands) administration allowed to conclude that the excessive ACTH response to stress exposure in old animals with DAB may be due to excessive activation of vasopressin V1b receptors on pituitary corticotrophs, and the use of antagonists of these receptors is promising for its prevention [80, 82].

**Figure 4:**
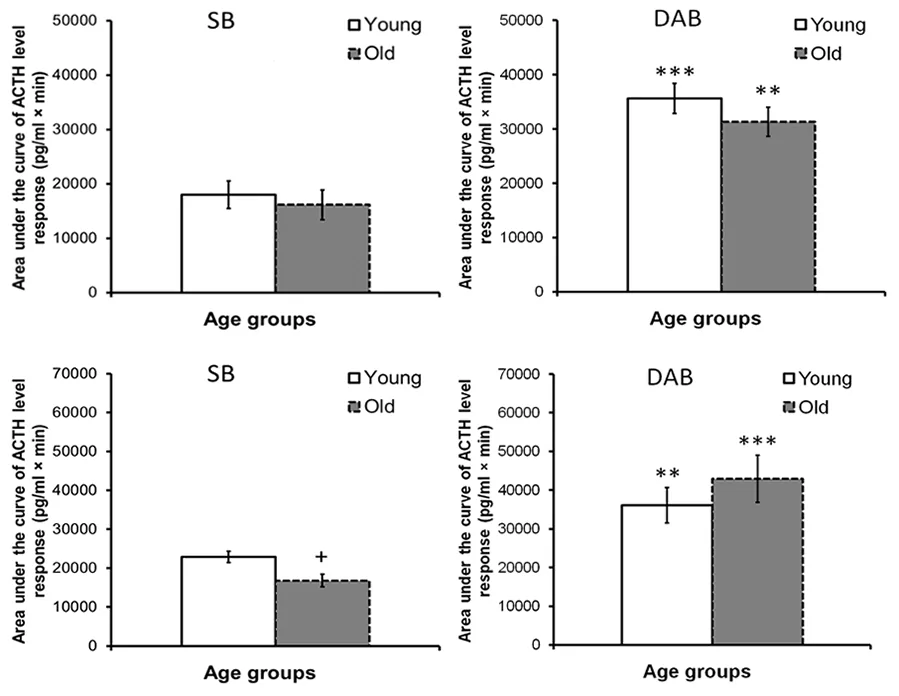
**The area under the curves of ACTH level response to functional tests with administration of CRH (at top) and AVP (at bottom) in young and old female rhesus monkeys with different types of behavior (mean±S.E.M; begun at 15:00h).** **p < 0.01; **p < 0.001 – vs. relative values in animals with SB; + p <0.05 – age-related differences.

Since the search for individual differences in the HPA axis response to ASE in female rhesus monkeys was conducted in the afternoon and evening time, the period of greatest sensitivity of the HPA axis to ASE [72, 78] and minimal circadian activity of the HPA axis [11, 70, 73, 76, 83], it could also be assumed that, with aging, intergroup differences in the regulation of the HPA axis by the negative feedback mechanism at the level of mineralocorticoid receptors (MR) are possible. Indeed, the functional test with Fludrocortisone (FlUD, agonist of MR) revealed the existence of pronounced intergroup differences in HPA axis response to FlUD, with lower sensitivity in old animals with DAB. The differences were mainly due to the slower rate of onset of the inhibitory effect of FLUD on CORT secretion compared to placebo in DAB-treated than SB-treated animals [31]. The consequence of intergroup differences in HPA axis response to FLUD was the revealed intergroup differences in the functioning of the HPA axis in old animals under basal conditions during the period of minimal circadian activity of the HPA axis (18:00–22:00) with higher levels of ACTH and CORT in animals with DAB, i.e. absolute hypercortisolemia develops in individuals with DAB [31].

The data obtained in the experiment on nonhuman primates are in good agreement with the results of a number of clinical studies, which detected MR transcripts in postmortem brains of depressed patients in lower amounts in the hippocampus and prefrontal cortex compared to non-depressed individuals [75, 84, 85, 86]. It has also been found that MR in the hippocampus is activated in patients with depression during a course of antidepressants [75, 84, 86], and stimulation of MR with FLUD decreases the secretion of CORT and improves cognitive function in individuals with depression [87]. As is known, the hippocampal MR plays a leading role in controlling the activity of the HPA axis in the evening in humans and nonhuman primates [14, 85, 88]. It is also possible that female rhesus monkeys with DAB contain a polymorphic form of the MR gene, which is more vulnerable to stress and the aging process. For instance, NR3C2 is found in a number of healthy people, as well as in patients with depression and other mental illnesses [85, 86]. In contrast to the results of the FLUD test, no significant intergroup differences were found in sensitivity of the HPA axis to dexamethasone, an agonist of glucocorticoid receptors, in old female rhesus monkeys with DAB and SB [31].

It should be emphasized that a number of changes in the functioning of the HPA axis during aging in primates with DAB described above are in good agreement with the results of studies of HPA axis function in adult people and animals exposed to severe early life stress (ELS). As shown by studies in humans and animals underwent childhood trauma, ELS induces long-term hyperactivity of the HPA axis, which is associated with changes in DNA methylation of various genes involved in the regulation of HPA axis activity, in particular, GR, FKBP51, CRH, AVP, pituitary proopiomelanocortin [66, 85, 89, 90], induce depression and other psychiatric disorders in humans [66, 85, 89, 91], promote to the development of mood disorders in nonhuman primates during adolescence [92, 93]. A stimulating effect of maternal deprivation experienced by female rhesus monkeys in neonatal period on the development of DAB behavior, as well as dysfunctions in the HPA axis in adulthood and aging, has been revealed [94].

Thus, it was revealed that there are pronounced age-related differences in the functioning of the HPA axis in monkeys with DAB compared to SB under basal conditions and under stress, such as smoothing of the circadian rhythm of CORT with an increase in its concentration at night, increased tone of the vasopressinergic system, impairment negative feedback regulation based on mineralocorticoid receptors with an increase in basal levels of CORT in the evening (18:00-22:00) in old animals with DAB. The latter were also characterized by a higher ACTH response and the CORT/DHEAS molar ratio to ASE, and a weakening of the sensitivity of the adrenal cortex to ACTH. In turn, hypercortisolemia, including relative (increase CORT/DHEAS molar ratio in response to ASE), which develops during aging in animals with DAB, can be an important pathophysiological factor in the development of dysfunctions of the HPT axis, the pancreatic islet apparatus, disorders of carbohydrate and lipid homeostasis, and contribute to the development of various aging-associated diseases, including mental, diabetes 2 type, neurodegenerative and others [8, 11–14, 40, 45, 77, 95]. The identified age-related disturbances in circadian rhythmicity in various aspects of HPA axis functioning in animals with DAB may be important for adequate adaptation to environmental stress, because almost all physiological effects of glucocorticoids from regulation of metabolic and immune signaling to effects on cognitive processes and host behavior, are exposed to similar rhythms showing a strong dependence on time of day and the close relationship between HPA axis activity and the circadian system [11, 70, 72, 73, 76, 83]. In addition, elevated CORT levels are negatively correlated with a decrease in life expectancy in humans [44] and survival in a wild primate population [96].

## Hypothalamic – pituitary - thyroid axis

The results of experimental studies of the features of the functioning of the HPT axis in the same female rhesus monkeys, in which individual and age-related features of the activity of the HPA axis were studied (see section above), made it possible to identify a number of significant individual differences in HPT axis functioning, which largely correlated with individual and age-related differences in the activity of HPA axis. Thus, thyrotropin (TSH) and free fractions of triiodothyronine (T3) and thyroxine (T4) in female rhesus monkeys exhibit circadian profiles in the circulation that are negatively correlated with the circadian profile of ACTH and CORT. Unlike ACTH and CORT plasma profiles, the minimum values of TSH and free fraction of T4 in young rhesus monkeys were detected in the morning hours (09:00) and the maximum values were detected at 22:00-03:00 [41, 54, 97]. In humans, the concentration of TRH, TSH, T3, and T4 demonstrated a nocturnal peak around 02:00–04:00 and the minimum during daytime [98–101].

The circadian rhythm of TSH in old rhesus monkeys, regardless of behavioral features, retained the same profile as in young female rhesus monkeys [54, 97]. At the same time, pronounced intergroup differences were revealed in the characteristics of age-related changes in the concentration of thyroid hormones. Thus, aging does not alter the levels of circulating T3 but decreases circulating levels of T4 in rhesus monkeys [102, 103, 104, 105, 106] and female western lowland gorilla (Gorilla gorilla gorilla) [104] or leads to a simultaneous decrease in both fractions of thyroid hormones as in females, as in males cynomolgus monkeys (*Macaca facsicularis*) probably due to the combination of altered sensitivity of the thyroid gland to TSH and decreased secretory activity of the thyroid gland [106]. Concentration of free T4 remained without significant changes with aging in female rhesus monkeys with SB, but statistically significantly decreased in old animals with DAB at 9:00, 15:00, and 22:00 [97]. The levels of free T3 fraction during aging in animals with SB statistically significantly decreased at 15:00, 22:00, and 03:00, but not at 9:00, and remained without significant changes in old animals with DAB [97]. The discussed age differences in the characteristics of thyroid hormone circadian rhythms in nonhuman primates with DAB and SB behavior are original and are illustrated in Figure 5.

**Figure 5:**
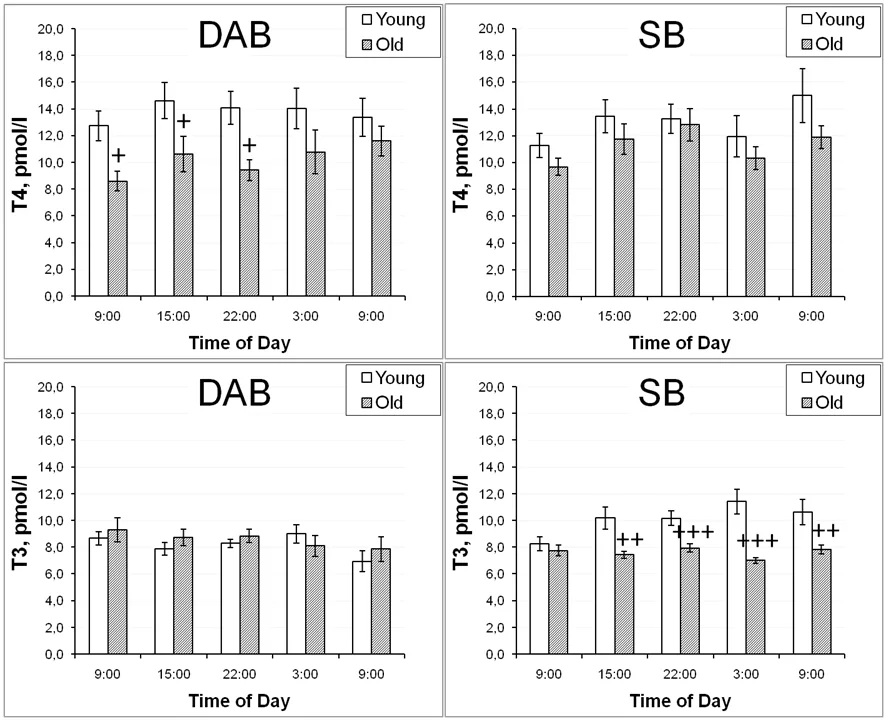
**Dynamics of free T4 and free T3 levels in peripheral blood plasma of young and old female rhesus monkeys with various types of behavior at different time of day (mean±S.E.M.).** + p<0.05, ++ p<0.01, +++ p<0.0001 vs. relative values in old animals.

A decrease in serum T3 levels with aging has been repeatedly observed in healthy people of both sexes [8, 101, 107]. A number of clinical studies have also noted a decrease in T4 secretion with age [18, 108, 109]. In addition, it was noted that in elderly individual subclinical hypothyroidism (increased TSH concentrations and normal levels of thyroid hormones in the blood serum) is more common than overt hypothyroidism [110–112].

The differences in the direction of age-related changes in thyroid hormone concentrations in primates with SB with a decrease in free T3, as well as the results of clinical studies that found a marked decrease in the plasma T3 levels of healthy aging people, are apparently due to a marked change in the activity of peripheral iodothyronine deiodinases. It is known that iodothyronine deiodinases in peripheral tissues and brain (iodothyronine deiodinase-1 and iodothyronine deiodinase-2) convert T4 to T3 and iodothyronine deiodinase-3 convert T3 to inactive reverse T3 (rT3) [98, 113]. It is possible that with aging in monkeys with SB, the activity of deiodinases is impaired in the afternoon and nighttime, which leads to a marked decrease in the concentration of free T3 not in the morning hours, but during the day and at night. Indeed, there is evidence in the literature that aging reduces the activity of iodothyronine deiodinase-1 and increases iodothyronine deiodinase-3 activity [114, 115].

In contrast to thyroid hormones, TSH levels tended to increase with age in rhesus monkeys of both behavioral groups (SB and DAB) [116]. Most clinical data also suggest that TSH levels tend to increase with age [110, 117, 118, 119, 120]. It should also be noted that there were statistically significant intergroup differences in basal TSH values in young animals with DAB and SB, with lower values in animals with DAB in the absence of significant intergroup differences in the concentration of thyroid hormones [116]. These intergroup differences in TSH levels in young monkeys may be due to a lower concentration of TRH receptors on the pituitary thyrotrophs in females with DAB, which in turn may be due to a decreased TRH secretion in the PVN of the hypothalamus. Thus, a marked decrease in mRNA expression for TRH was detected in pathological material from patients with depression [121].

The rhesus monkeys with SB and DAB differed not only in age-related features of the functioning of the HPT axis under basal conditions, but also in their response to the administration of specific activators of thyroid function (TRH and TSH). Thus, for animals with DAB, more pronounced age-related disturbances in the HPT axis response to the functional test with TRH were revealed [116]. They were characterized by a statistically significant decrease in the magnitude of the rise in the concentration of free T4 and a pronounced increase in the magnitude of the rise in the concentration of TSH in the absence of significant age-related changes in the response of free T4 and TSH in monkeys with SB. In contrast to the free T4 and TSH secretion, no significant age-related differences in the response of free T3 to this test neither in animals with DAB, nor in animals with SB were found [116]. The opposite changes in the T4 and TSH reactions in response to the TRH test in old monkeys with DAB indicated a possible damage to the cells of the thyroid gland itself, including a weakening of sensitivity of thyroid thyrocytes to TSH. Indeed, an additional functional test with TSH administration to female rhesus monkeys with SB and DAB of both age groups revealed a lower secretory response of the thyroid gland in old animals with DAB compared to young animals with the same behavioral type. At the same time, in animals with SB, no significant age-related differences were found in the response of thyroid hormones to the TSH test [97]. Figure 6 shows age-related differences in the response of free T4 to TSH administration in female rhesus monkeys with DAB and SB. These data suggested that with aging, DAB females develop primary dysfunction of thyrocytes, leading to a decrease in the secretion of free T4 and, through a negative feedback mechanism, to the activation of the hypothalamic-pituitary axis. Similar age-related changes in the functioning of the HPT axis were also noted by other authors in an experiment on the cynomolgus monkeys of different ages without taking into account the peculiarities of their adaptive behavior [103].

**Figure 6:**
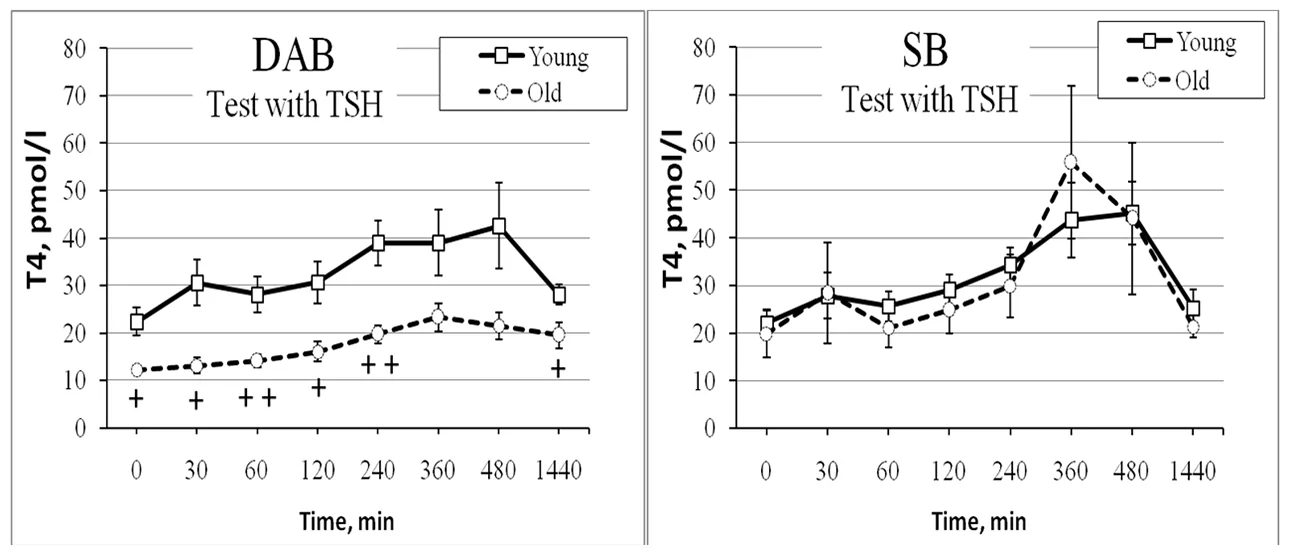
**Dynamics of free T4 level in peripheral blood plasma of old female rhesus monkeys with different types of behavior in response to administration of TSH at 09:00h (mean±S.E.M.).** + p < 0.05; ++ p <0.01– vs. relative values in young animals.

The revealed differences in the functioning of HPA and HPT axes in old rhesus monkeys with DAB compared to healthy SB monkeys give reason to believe that there are differences in age-related changes of metabolic processes in these behavioral groups, since, as already indicated above (see sections: Introduction, Hypothalamic-pituitary-adrenal axis), thyroid and steroid hormones regulate lipid and carbohydrate metabolism, and also affect the function of the islet apparatus of the pancreas [8, 17–22, 29, 45, 77, 95, 97, 122–125]. Indeed, a positive correlation was found between thyroid activity (plasma free T4 level) and body mass index in young monkeys and a negative correlation in old ones, regardless of behavioral characteristics [116]. An increase in triglyceride concentrations with age was observed in both DAB and SB female rhesus monkeys, regardless of body weight [79, 97]. However, age-related changes in triglyceride concentrations in overweight animals were behaviorally dependent: in old animals with DAB, triglyceride levels were lower than in old animals with SB [79, 97]. More pronounced indicators of insulin resistance and impaired insulin secretion were also noted in old female rhesus monkeys with DAB and overweight compared to old monkeys with SB and overweight [37]. Apparently, a deficiency of compensatory insulin secretion in old DAB monkeys with overweight, which is known to stimulate the synthesis of triglycerides and fatty acids, underlies the identified phenomenon with triglyceride levels. In turn, triglycerides and fatty acids are used by cells as energy material in severe insulin deficiency [19]. It should also be noted that, along with more pronounced disorders in age-related changes in the functions of neuroendocrine systems and the metabolism of lipids and glucose, the female rhesus monkeys with DAB showed the greatest age-related disturbances (decrease) in the activity of the antioxidant enzyme defense system and the activation of lipid peroxidation process, one of the well-known biomarkers of acceleration aging and age-related pathology [33, 126].

The results of experimental studies on nonhuman primates are in good agreement with the clinical trial data [108, 111, 112, 115, 127, 128]. Thus, it was demonstrated that patients with overt hypothyroidism have a higher chance of developing type 2 diabetes [18, 115, 125, 129, 130]. Subclinical hypothyroidism increases insulin resistance in normoglycemic people [131]. Obesity is an important factor not only in insulin resistance of various tissues, but also in the long-term increase in the secretory capacity of the β-cells of the pancreas [127]. A clear correlation between body mass index and β-cell mass was shown in humans [128]. In addition, it has been repeatedly noted that dysfunctions of both the HPT axis and the pancreas are closely associated with obesity [19, 122, 127, 132, 133]. At the same time, the association between low thyroid function and an increased risk of diabetes and cardiovascular pathology was noted [16, 20, 111, 112, 115, 132].

Thus, with aging changes in the functioning of the HPT axis develop in primates, the features and severity of which depend on the psycho-physiological characteristics of the individual, with more pronounced disturbances in animals with DAB. Old animals with DAB develop hypothyroxinaemia in the morning, evening, and night time in the absence of significant changes in the concentration of free T3. At the same time old animals with SB develop a decrease in plasma T3 during the day and night hours, a period of increased circadian thyroid activity. Age-related changes in the HPT axis function in primates are accompanied by pronounced changes in lipid metabolism, glucose homeostasis, and insulin secretion, correlating with the characteristics of adaptive behavior and body weight of the individual. More pronounced metabolic disturbances are characteristic of overweight animals with DAB. The results of studies in nonhuman primates may underlie the inconsistency of the results of clinical studies examining the function of the HPT regardless of the adaptive behavior of individuals (their psychophysiological characteristics), who did or did not observe a decline in thyroid function with aging and they are consistent with the results of clinical studies that have revealed different trajectories of metabolic, cognitive, and mental aging [2–4, 6, 12] and in light of new data on the regulatory influence of thyroid hormones on lifespan through modulation of key cellular pathways associated with longevity and the main hallmarks of aging [15]. The data on the decrease in the level of free T3 in the blood plasma of old monkeys with SB not in the morning hours, but in the afternoon - night time may have important practical significance, since they indicate the prospects of the procedure for collecting blood samples in the diagnosis of thyroid dysfunction in the elderly not only at 9:00, but also at other times of the day, for example, at 15:00 or 22:00.

In conclusion, changes in the demographic situation with an increase in the proportion of elderly and senile people, and an expansion of the range of stressful influences have caused a sharp increase in the incidence of age-related socially significant diseases (psychiatric, metabolic, endocrine, cognitive, cardiovascular, neurodegenerative) in the etiopathogenesis of which an important role is given to dysfunctions of HPA and HPT axes, the most important adaptive neuroendocrine axes. Clinical studies demonstrate the existence of distinct trajectories of cognitive and metabolic aging. Experimental studies highlight the importance of steroid and thyroid hormones in regulating key cellular pathways associated with longevity and the main hallmarks of aging [15]. Of significant interest in this regard are also physiological studies conducted in experiments on primates, the best translational model, aimed at finding biomarkers of increased vulnerability and resistance of the body to stressful influences and identifying the characteristics of stress exposure in old and senile age. Among them, studies on the study of individual characteristics of changes in the function of HPA and HPA axes and a number of metabolic indicators in primates at different age periods using medical functional tests are quite informative and of significant scientific and practical interest. The use of diagnostic tests has revealed a number of indicators that can be used to identify individuals predisposed to the development of age-related pathology at a young age: DAB behavior under mild stress, a history of severe stress in early childhood, an increased ACTH response to CRH and AVP, reduced basal TSH levels, excess body weight, and signs of impaired glucose tolerance. A deeper understanding of the age-related changes in neuroendocrine function and their role in aging pathways may provide information on new strategies to promote healthy aging, including modulation of thyroid and adrenal hormones and personalized endocrine optimization. Monitoring the behavior of individuals, as well as the functions of key adaptive endocrine systems, is promising for the early diagnosis of age-related pathology, its prevention and personalized treatment.
